# A Variant of Crossed Pulmonary Arteries in Association with Coarctation of Aorta

**DOI:** 10.7759/cureus.2477

**Published:** 2018-04-13

**Authors:** Rabail Raza, Kumail Khandwala, Hafsa Qayyum, Muhammad Anwar Saeed, Anwar Ahmed

**Affiliations:** 1 Department of Radiology, The Aga Khan University, Karachi, PAK; 2 Department of Radiology, Rashid Hospital, Dubai, ARE

**Keywords:** crisscross, crossed pulmonary arteries, computed tomography, coarctation of aorta, down syndrome

## Abstract

Crossed or "crisscross" pulmonary arteries (CPA) are a result of an anomalous origin and course of both the pulmonary arteries from the main pulmonary trunk in which the left pulmonary artery (PA) ostium usually lies directly superior and to the right PA ostium after which they cross each other and supply their respective lungs. This condition is usually associated with conotruncal malformations and genetic syndromes. We describe a case report of an infant, suspected to have Down syndrome, who was diagnosed with CPA and coarctation of aorta on computed tomography (CT) angiography. Our case is unusual because in our patient the right PA ostium was superior and to the left of the left PA suggesting a variant of CPA that has not been documented before. Three-dimensional (3D) CT reconstruction has shown to improve the understanding of this anomaly and its unique 3D display of various angles may enhance anatomical comprehension of such complex cases.

## Introduction

Crossed pulmonary arteries (CPA) refer to an anomalous origin of both pulmonary arteries from the main pulmonary trunk. The pattern of “crisscross pulmonary arteries” is a rare classic malposition of the branch pulmonary arteries in which they cross each other and supply the respective lungs. It was first described by Jue et al. in 1966 [1]. Historically it has been described that in this condition the left pulmonary artery (PA) ostium usually lies directly superior and to the right of the right PA ostium. This anomaly is often associated with conotruncal malformations and genetic syndromes, including trisomy 18 and 22q11 deletions. One report by Chen et al. suggested that fewer than 39 cases had been reported up to 2013 [2].

We describe a case report of an infant who was suspected to have Down syndrome and was diagnosed with CPA and coarctation of aorta on computed tomography (CT) angiography. Our case is unusual because in our patient, the right PA ostium was superior and to the left of the left PA, suggesting a variant of CPA that has not been described before to the best of our knowledge. We aim to share the radiological findings with a particular emphasis on computed tomography angiography imaging and three-dimensional reconstruction techniques.

## Case presentation

A two-month-old male child presented to the emergency department with complaints of reluctance to feed, cough and respiratory distress for one week. On clinical examination, the child was found to be pale looking and irritable, with dysmorphic Mongolian facies, single palmar crease, and broad thumbs. These features were suggestive of Down syndrome. Cardiovascular examination revealed S1 + S2 heart sounds with a Grade 3 pansystolic murmur at the left sternal border. Bilateral coarse crepitations were audible on respiratory examination. His family was from a rural background and, therefore, his prior medical history was not remarkable, although his mother claimed he was generally well before the onset of symptoms. Considering the presenting symptoms and clinical examination, it was decided to perform further investigations.

**Figure 1:**
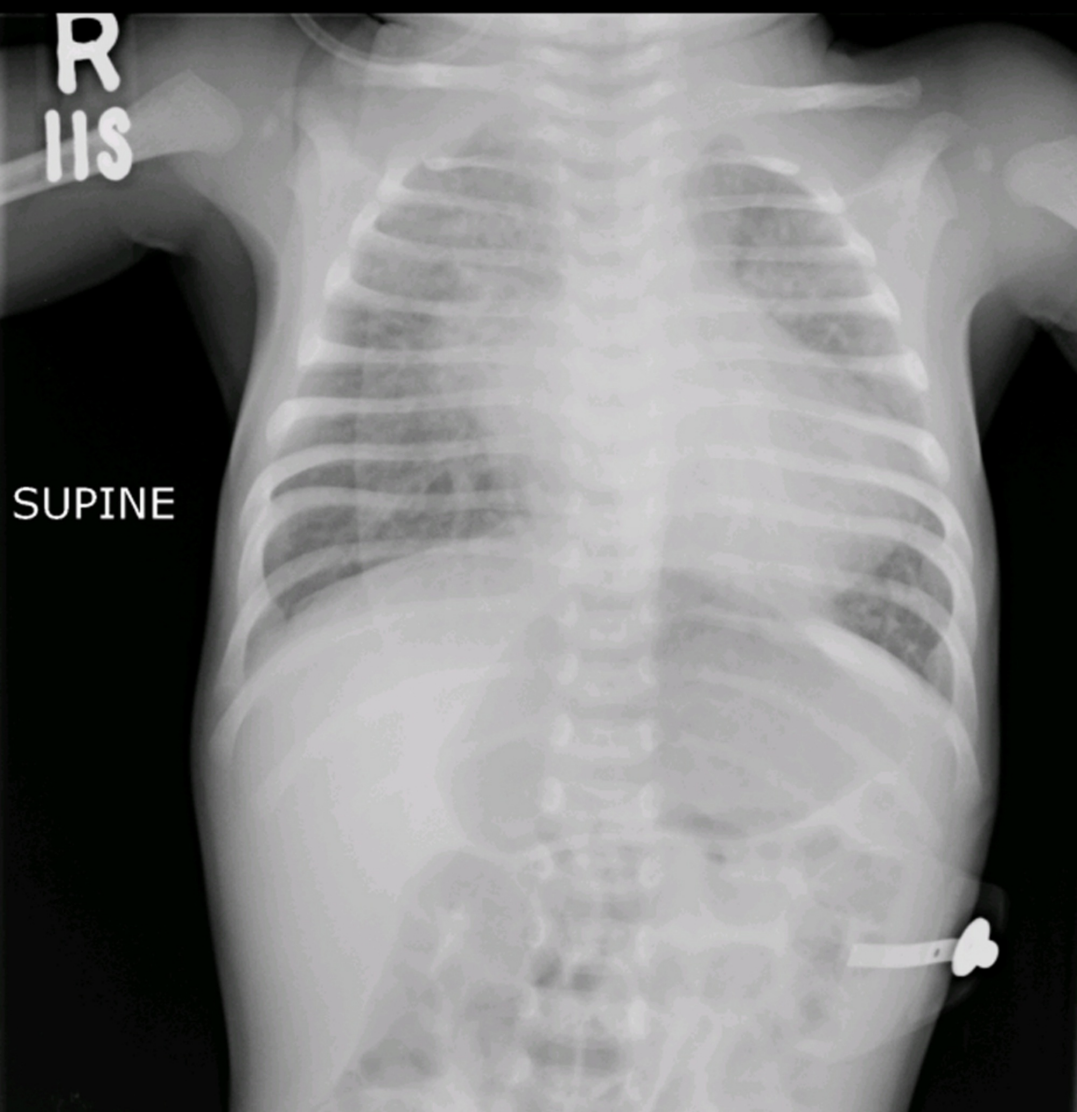
Chest X-ray Rounding and elevation of the cardiac apex along with pulmonary vascular congestion, suggestive of congenital heart disease with pulmonary plethora.

An initial chest X-ray showed rounding of the cardiac apex with pulmonary plethora suggesting congenital heart disease (Figure 1).

Echocardiogram findings included presence of large ventricular septal defect (VSD), double outlet right ventricle (DORV), small patent ductus arteriosus (PDA), coarctation of aorta, left ventricular hypertrophy, and left ventricular diastolic dysfunction (LVDD). The bifurcation of pulmonary arteries was not clearly demonstrated.

**Figure 2:**
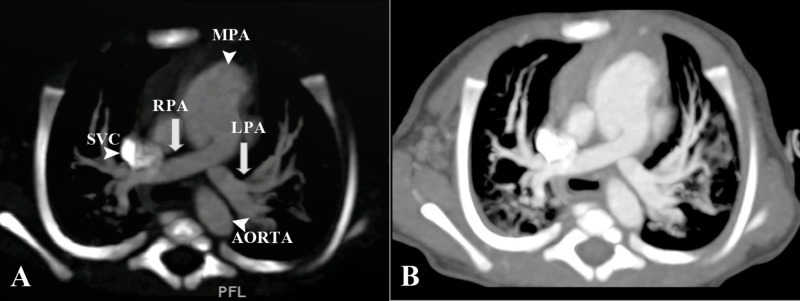
CT cardiac angiography axial sections “Crisscross” pattern of right pulmonary artery (RPA) and left pulmonary artery (LPA) (white arrows). Also labelled are the main pulmonary trunk (MPA) superior vena cava (SVC) and descending aorta (arrowheads).

Computed tomography (CT) cardiac angiography was then performed which showed crisscross pattern of the right and left pulmonary arteries from the main pulmonary trunk (Figure 2).

**Figure 3:**
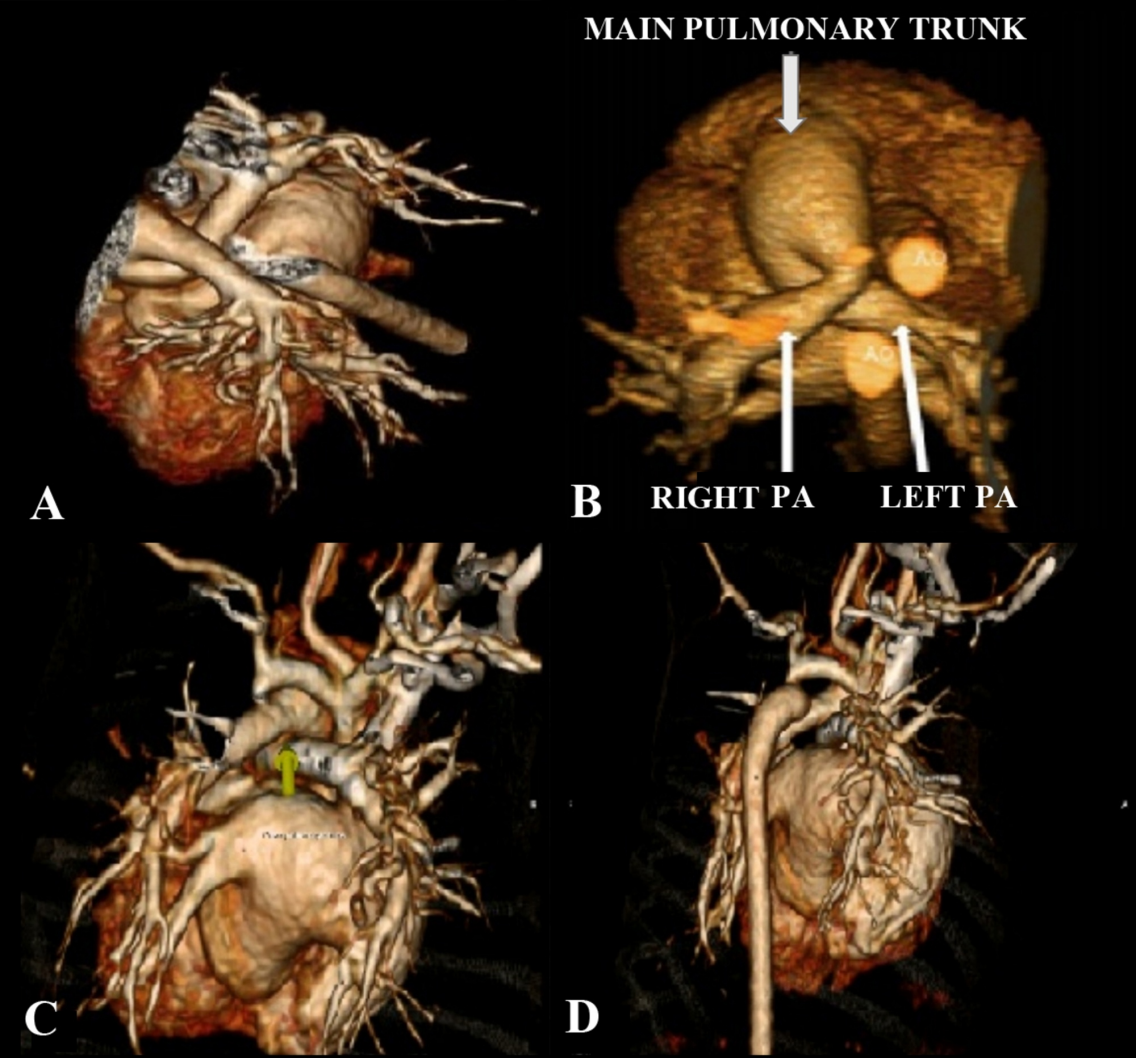
Three-dimensional reconstruction images 3D anatomical configuration of the crossed pulmonary arteries. PA: pulmonary artery

This was evident by the right pulmonary artery arising superiorly and to the left side of the left pulmonary artery, after which it was crossing the midline to supply the respective lung. The left PA had a vice versa course. The crossed pattern was causing main pulmonary trunk dilatation. These findings and relationships of the PA ostia were further confirmed by three-dimensional (3D) reconstructions (Figure 3).

**Figure 4:**
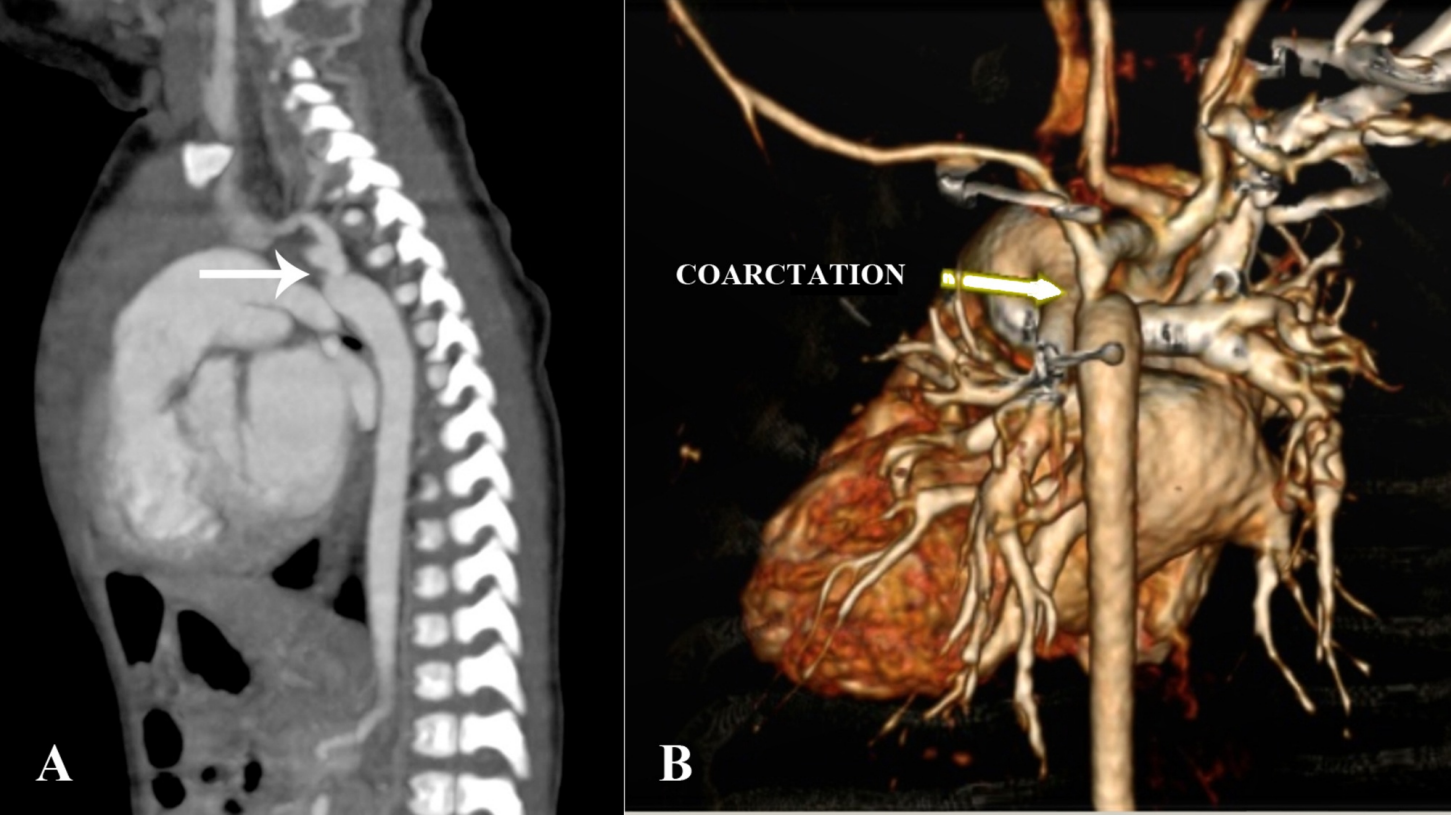
CT angiography sagittal (A) and 3D reconstruction (B) images Post-ductal coarctation of aorta (arrows) distal to the origin of the left subclavian artery.

There was also post-ductal aortic coarctation with significant narrowing just distal to the origin of left subclavian artery (Figure 4).

A perimembranous VSD with DORV was also noted; however, the small PDA could not be clearly demonstrated on CT. Atrioventricular concordance was noted with normal drainage of four pulmonary veins into the left atrium and superior and inferior vena cava draining into the right atrium. No anomalous pulmonary venous return or significant aortopulmonary collateral filling was seen. There was no airway compromise or vascular sling and the pulmonary arteries were crossing anterior to the trachea.

Initially, the patient was managed along the lines of a respiratory tract infection and was discharged in a stable condition. Two months later, he was electively admitted for surgery. A two-stage procedure was carried out for coarctation repair and VSD closure. Intra-operatively, the patient developed complications including hypertension, mixed respiratory acidosis, and bradycardia. Post-operatively the patient was shifted to the intensive care unit with an open chest and was kept on ventilation. Intermittent respiratory distress and bradycardia complicated his post-operative course and despite inotropic support, he died on the eighth day after surgery.

## Discussion

In CPA, typically the pulmonary arteries cross each other and supply the respective lungs. The left PA ostium usually lies directly superior and to the right PA ostium, however in our case the anatomy was vice versa suggesting a variant of CPA that has not been documented before. The etiology of this condition has not been established, but it is thought that the crisscross origin results from either faulty differential growth during partitioning of the truncus arteriosus or abnormal rotation of the aortic and pulmonary trunks, usually as a result of counterclockwise rotation of the normal origins of the branch pulmonary arteries. Therefore, in our case we propose that a clockwise rotation has most likely resulted in an opposite orientation of the crossed pulmonary arteries. In most reported cases, this anomaly is diagnosed in association with truncus arteriosus and interrupted aortic arch, VSD, ASD, tetralogy of Fallot, and left superior vena cava [3-5].

Post-ductal aortic coarctation, double outlet right ventricle, and small PDA were discovered in our patient. These associations have also previously been reported by Zimmerman et al in a review of six cases of crossed pulmonary arteries [5]. In spite of the anomalous location and aberrant course of the pulmonary arteries, the crossed vasculature does not cause hemodynamic abnormalities or airway obstruction, and therefore by itself, this condition is generally considered benign. However, in instances when pulmonary pressure exceeds the systemic pressure, right to left shunting across prevailing cardiovascular channels like the PDA occurs, which causes systemic hypoxemia and cyanosis [6].

Airway compromise, when present, is usually related to extrinsic vascular compression by either dilated branch pulmonary arteries, by a malpositioned descending aorta or by a pulmonary arterial sling. The differentiating feature between pulmonary arterial sling and crisscross pulmonary arteries is that the left pulmonary artery courses between the trachea and the esophagus in pulmonary sling, whereas the pulmonary arteries cross anterior to the trachea in CPA [7]. This was also observed in our case.

Echocardiography, conventional and computed tomography cardiac angiography and magnetic resonance imaging (MRI) have all been used as modalities to diagnose crisscross pulmonary arteries [5, 8-9]. The diagnosis is usually quite difficult on echocardiography. However, if the bifurcation of the pulmonary arteries is not clearly demonstrated, this may be a clue to making the diagnosis. MRI makes use of the benefits of multiplanar and multi-sequential imaging, but relies on lengthy acquisition times, therefore limiting its application in unstable patients. Dual-source CT angiography may be effective for diagnosis due to high spatial resolution and short scan time, which ultimately decreases the need for sedation and general anesthesia. Three-dimensional reconstruction greatly improves the understanding of this anomaly and unique 3D display of various angles may enhance comprehension of complex anatomies it is associated with. It also allows preoperative management and simulation as well as postoperative evaluation. However, the use of ionizing radiation and the risks of administering iodinated contrast agents have been some of the disadvantages of CT [10].

Since CPA is a condition that in itself is generally considered benign, it does not warrant surgical correction. However, in the presence of other complex cardiac anomalies or right to left shunting, surgical intervention may be indicated. Surgical correction was attempted for VSD closure and repair of aortic coarctation in our patient which proved to be fatal. The prognosis of crisscross pulmonary arteries is therefore relatively good unless it is accompanied by other structural cardiac or genetic abnormalities.

## Conclusions

In conclusion, we report a case of an infant, suspected to have Down syndrome, who was diagnosed with crisscross pulmonary arteries and post-ductal aortic coarctation on CT angiography. Our case appears to be the first variant of CPA in which the right PA ostium was lying superior and to the left of the left PA ostium, probably secondary to an abnormal clockwise rotation instead of the usual counterclockwise rotation that has been proposed in literature. 3D reconstruction images are useful for visualizing this condition and its associations, and may also help in preoperative simulation and management. The most important diagnostic feature of crisscross pulmonary arteries is to disclose the exact relationship between the left and right pulmonary arteries and their ostia as such variants may exist. This condition is usually accompanied with other congenital heart diseases and extracardiac anomalies, and is therefore important to consider in the list of differential diagnoses of pulmonary artery abnormalities in genetic syndromes.
